# Recent Advancements in Humanoid Robot Heads: Mechanics, Perception, and Computational Systems

**DOI:** 10.3390/biomimetics10110716

**Published:** 2025-10-22

**Authors:** Katarina Josic, Maysoon Ghandour, Maya Sleiman, Wen Qi, Hang Su, Naima AitOufroukh-Mammar, Samer Alfayad

**Affiliations:** 1 KALYSTA Actuation, 91000 Evry, France; 20235685@etud.univ-evry.fr; 2 The IBISC Laboratory, University of Evry Paris-Saclay, University of Paris-Saclay, 91000 Evry, France; maysoon.ghandour@gmail.com (M.G.);; 3 School of Future Technology, South China University of Technology, Guangzhou 511442, China

**Keywords:** humanoid head, review, mechanics, perception, computational systems, artificial intelligence

## Abstract

This paper presents a comprehensive review that provides an in-depth examination of humanoid heads, focusing on their mechanics, perception systems, computational frameworks, and human–robot interaction interfaces. The integration of these elements is crucial for developing advanced human–robot interfaces that enhance user interaction and experience. Key topics include the principles of context, functionality, and appearance that guide the design of humanoid heads. This review delves into the different aspects of human–robot interaction, emphasizing the role of artificial intelligence and large language models in improving these interactions. Technical challenges such as the uncanny valley phenomenon, facial expression synthesis, and multi-sensory integration are further explored. This paper identifies future research directions and underscores the importance of interdisciplinary collaboration in overcoming current limitations and advancing the field of humanoid head technology.

## 1. Introduction

Artwork throughout history, such as the Venus of Willendorf sculpture [1] and the stories of the Golem from the 16th century [2], demonstrates our constant search for ways to distill essential human traits and create artificial life that represents who we are.

**Figure 1:**
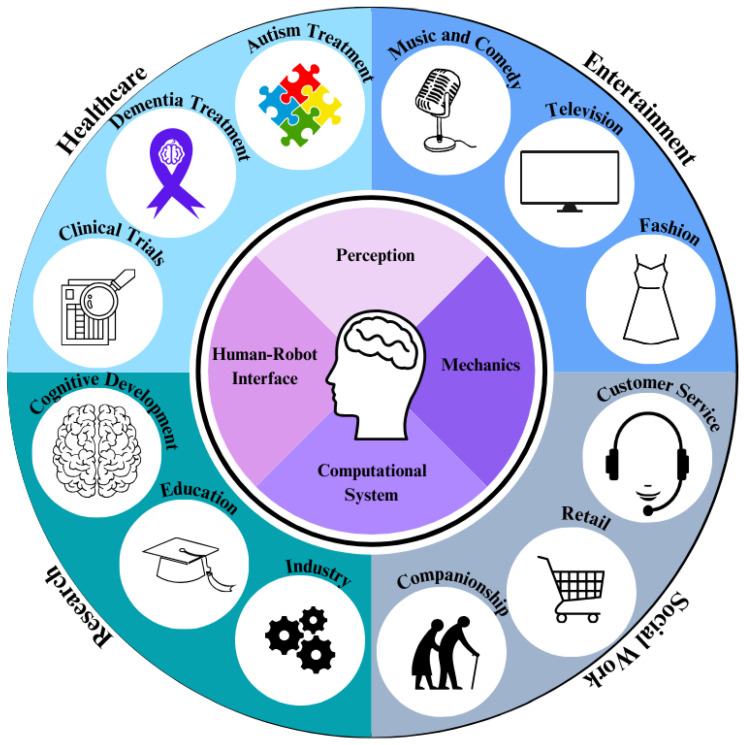
Overview of humanoid robot head applications.

Emulating human-like behavior in artificial agents can activate social cognitive mechanisms to a degree comparable to interactions with human partners. To enhance the social competence of such agents, it is therefore effective to employ an interface that people find relatable [3,4], such as a humanoid head. The guiding principles behind humanoid head design are summarized in Figure 1. Building on this ideology, humanoid robots have been developed via human–robot interfaces in the form of a head, combining appearance, perception, and communication functions in a single focal point.

Even though humanoid robotics has made significant strides, enabling robots to interact with people naturally and comfortably remains challenging [5]. The humanoid head is central to this goal: it confers a human-like appearance and serves as the multimodal interface for perception and communication. A competent head should render facial expressions as paralinguistic signals for nonverbal communication and act as a sensing hub that acquires data for perception, navigation, and dialogue [6]. In this paper we adopt three working principles for defining a humanoid robot head: context (the head is designed for integration into a humanoid robot), functionality (it provides at least one primary head-mounted sense, e.g., vision or audition), and appearance (it resembles a human head or reliably elicits anthropomorphic perception).

**Figure 2:**
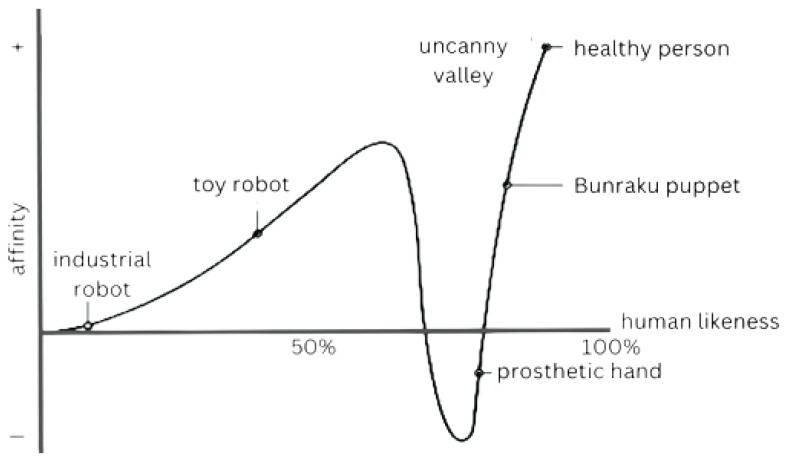
The uncanny valley: The relationship between an entity’s human-likeness and the observer’s affinity.

However, achieving a seamless human-like appearance is constrained by the uncanny valley [7], the theorized relation between an entity’s human-likeness and the observer’s affinity [8], shown in Figure 2. In practice, the left local maximum typically corresponds to stylized or clearly robotic heads that avoid perceptual mismatches—for example, toy-like or cartoon designs such as NAO, Kibo, or Zeno. The local minimum (the valley) appears for almost-human embodiments, where subtle temporal or material inconsistencies (skin reflectance, blink latency, ocular micro-saccades, speech–lip synchrony) trigger discomfort; highly realistic android heads such as Geminoid F or Sophia exemplify this regime. Crossing to the rightmost rise requires not only morphological realism but also tightly controlled micro-movements and temporally aligned multi-sensory cues.

**Figure 3:**
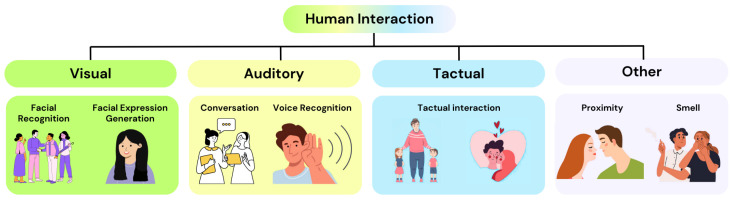
Aspects of interpersonal communication.

Technical constraints reinforce these perceptual limits: producing believable facial expressions, synchronizing prosody with visemes, coordinating gaze and blinks, and modeling affect all tax actuation, sensing, and control. Integrating these elements into a coherent, robust, and safe system further raises challenges of cost, durability, and energy efficiency. While some senses could be placed elsewhere on the body, head-mounted vision and audition are essential for human-like interaction and proxemics. By mimicking key cues of interpersonal communication (Figure 3), the perceptual abilities of a humanoid head can make human–robot interaction more comfortable and effective.

In comparison to other review articles [5,6] on humanoid heads, this review introduces several unique contributions. While the existing literature often focuses on individual components such as sensory systems in isolation, this review offers a comprehensive analysis that integrates vision sense, with an emphasis on stereo vision systems and auditory and proprioceptive senses. The role of artificial intelligence (AI) and large language models (LLMs) in enhancing humanoid head functionalities is also investigated in this review. Furthermore, an in-depth examination of the computational systems, including operating systems, middleware, central processing units, communication protocols, and power systems, is conducted. Additionally, this review uniquely explores the applications and market dynamics of humanoid heads, offering insights into available solutions and their costs across several countries.

Section 3 examines the current and potential uses of and demand for humanoid robot heads in various industries. The mechanical aspects of humanoid robot heads are discussed in Section 4. Section 5 covers how these robots interpret and interact with their environment. Section 6 focuses on the computational systems that drive humanoid robots. Section 7 illustrates the critical impact of artificial intelligence on the development and capabilities of these robots. Finally, Section 9 summarises the key findings and suggests directions for future research.

## 2. Methodology

This review surveys humanoid robot heads (HRHs) that integrate mechanical structure, perception, and computational/control stacks for human–robot interaction. Prior surveys over the past five years have mainly focused on mechanical design [5] and sensing [6]; a consolidated treatment that also synthesizes control architectures and computational systems is still missing. We therefore conducted a structured review that combines curated databases with the peer-reviewed literature and major robotics proceedings, with search criteria displayed in Table 1.

**Figure 4:**
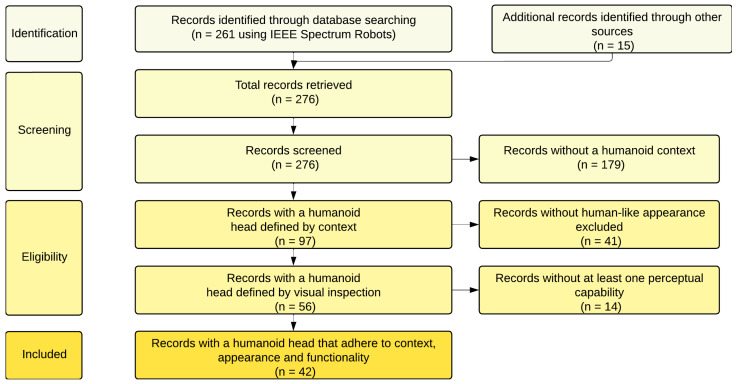
The PRISMA flow diagram for this systematic review of humanoid heads.

**Figure 5:**
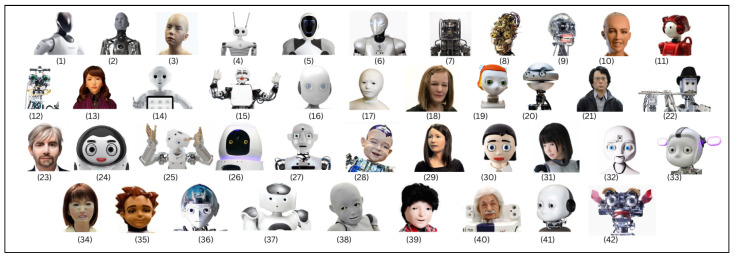
Database of humanoid robot heads in descending chronological order: (**1**) CyberOne, (**2**) Ameca, (**3**) Abel, (**4**) Reachy, (**5**) EVE, (**6**) Surena 4, (**7**) HRP-5P, (**8**) Na’vi Shaman, (**9**) Mesmer, (**10**) Sophia, (**11**) Emiew 3, (**12**) Tino, (**13**) Erica, (**14**) Pepper, (**15**) KOBIAN-RII, (**16**) Roboy, (**17**) Telenoid R4, (**18**) Nadine, (**19**) Dreamer, (**20**) Flash, (**21**) Geminoid HI-2, (**22**) Waseda Flutist, (**23**) Geminoid-DK, (**24**) Kibo, (**25**) M1, (**26**) Olivia, (**27**) RoboThespian, (**28**) Diego-san, (**29**) Geminoid F, (**30**) Flobi, (**31**) HRP-4C, (**32**) Octavia, (**33**) Simon, (**34**) Saya, (**35**) Zeno, (**36**) Kojiro, (**37**) NAO6, (**38**) CB2, (**39**) Kaspar, (**40**) Albert Hubo, (**41**) iCub, (**42**) Kismet.

We used the IEEE Spectrum Robots guide as a starting point [9], but—acknowledging the limitations of a single source [10]—extended the search to scholarly databases and conference proceedings. The review covers January 1998–December 2022, with the final search completed on 31 December 2022. Selection followed PRISMA guidelines (Figure 4). We first screened for contextual relevance (humanoid or android heads), then applied a visual appearance filter (human-like head), and finally enforced a functionality criterion (at least one head-mounted perceptual modality). This pipeline yielded the 42 HRHs analyzed here (1998–2022; see Figure 5). Figure 5 not only lists the final set of heads but also highlights their chronological distribution, from early experimental prototypes in the late 1990s to more recent socially interactive or commercial designs, providing temporal context for the trend analyses. For each head we extracted degrees of freedom, materials/skin, perceptual modalities, compute hardware and OS/middleware, communications/power, HMI, application domain, and (where available) cost; two reviewers coded each record and resolved disagreements by consensus. Inevitably, some recent or less-commercial platforms and price data were incomplete; we report ranges and explicitly note missing values where relevant.

## 3. Application and Marketplace

Humanoid heads have a variety of applications, including healthcare, social work, and entertainment. This section outlines different domains where humanoid heads are used and current market trends.

### 3.1. Healthcare and Communication

Robots with human-like appearances are expected to perform a range of human tasks, such as cognitive therapy, personal assistance, and senior social care [11]. Humanoid robots have been used to improve the motivation of healthy older adults to exercise and to help people with everyday tasks so they can live better lives. Having a humanoid head is essential for these kinds of jobs because the robot can interact with people in a non-intrusive way by speaking, making gestures, and expressing emotions [11].

Emiew 3 [12,13] and Pepper [14] are transforming customer service interactions in retail, hospitality, and at home. Flobi [15,16], M1 [17,18], and Geminoid F [19,20,21,22] contribute to communication media, and EVE [23] is designed to work alongside people. Nadine [24,25,26,27], Olivia [28,29], iCub(as of recently) [30,31,32], and Telenoid R4 [19,20,33,34] provide invaluable telepresence capabilities in social work and communication platforms, allowing for remote interactions and assistance. Diego-san [35,36] and CB2 [37,38,39] contribute to cognitive development research. Zeno’s [40,41] involvement in clinical trials and Pepper’s assistance [42] to people with dementia highlights their importance in medical research, providing invaluable assistance and support. Because of their programming, robots are accurate in every interaction, using the same gestures and facial expressions. The key to assisting young children with autism in learning the various facial expressions and gestures people use to convey their emotions, according to researchers, may lie in this consistency. Robots also play on the inclination of autistic children to favor predictable things [43]. Reachy [44,45,46], NAO6 [47,48,49,50], Kaspar [51,52,53,54], and Abel [55,56] have proven to be of great help in autism clinical trials.

### 3.2. Research Platforms

In the realm of education and research, all humanoid robot heads serve as platforms for discovering further development points. Kismet [57,58] was one of the first research platforms for humanoid heads. Surena 4 [59], Simon [60,61], Erica [19,20,34,62], and Saya [63,64,65,66] made significant contributions to development of humanoid heads. Flash [67,68], Roboy [69], Tino [70,71], Geminoid-DK [20,21,22,34,72], Kojiro [73,74], and Dreamer [75,76] should be mentioned as valuable assets to research. Ameca’s [77] adaptable design is useful in research, as well as product testing, reception and entertainment.

### 3.3. Entertainment

Entertainment venues rely on lifelike performances by humanoid robots, and humanoid heads have a positive effect on audiences. Na’vi Shaman [78] is distinguished by an interesting appearance and fluid movements, making her a great addition to Walt Disney World. Kibo [79,80] has a cute cartoonish face. Mesmer [81,82], Albert Hubo [83,84], and RoboThespian [85] enhance audience experiences. Waseda Flutist [86,87,88] uses music to help people and entertain them, whereas KOBIAN-RII [89,90,91] contributes humor and assistance in comedic roles. HRP-4C [92,93] serves in fashion entertainment. Sophia [94,95] is the world’s first robot citizen, having received Saudi Arabian citizenship, and she is a global name, having made appearances at worldwide conferences as well as on daytime TV shows in many countries.

### 3.4. Other Applications

In industrial settings, the humanoids CyberOne [96,97] and HRP-5P [98,99] help to streamline operations and ensure safety. Octavia [100,101] is responsible for firefighting. NAO6 [47,48,49,50] is useful for inspection and maintenance in industrial environments. Despite having defined humanoid heads, these applications do not necessarily require a humanoid head for their core tasks. The application of these humanoids is essentially to relieve human workers of tedious and hazardous tasks. However, to extend their applications to ones that require more HRI, such as customer support, in the future, as well as being a research platform, having a such head is beneficial.

### 3.5. Commercial Study

**Figure 6:**
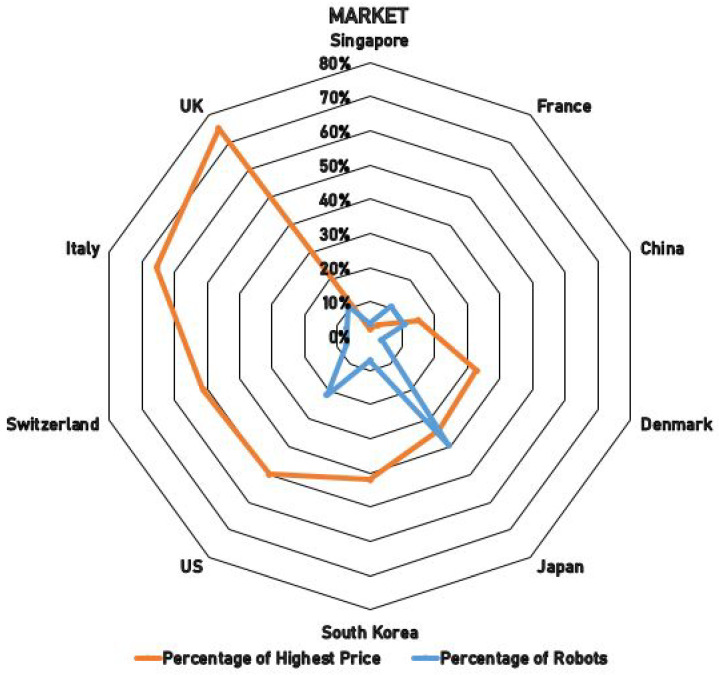
Comparison of the number of robots and the average cost of humanoid heads across countries.

To gauge market demand, we compared, by country of manufacture, (i) the number of humanoid heads represented in our dataset and (ii) the average list price of the models with available pricing; the results are summarized in Figure 6. Humanoid heads from Germany (Flobi [15,16]), Poland (Flash [67,68]), Iran (Surena 4 [59]), and Norway (EVE [23]) were also analyzed; however, due to missing price information, they are excluded from the price averages reported here.

Among countries with price data, Singapore shows the lowest observed average in our set, with the most affordable unit being Nadine [25,26,27] at approximately EUR 9,175. France also exhibits comparatively low prices, with an average around EUR 18,000. Japan contributes the largest sample (11 heads), with an average price of about EUR 156,521, while the United States is the second-largest contributor (6 heads), with an average of roughly EUR 228,635. The United Kingdom has the highest average price at approximately EUR 344,000. The single highest recorded price in our dataset is EUR 458,000 for Roboy [69] (Switzerland).

These figures should be interpreted with care: averages were only computed across models with available list prices and thus reflect coverage differences across countries. All amounts are reported in Euros; where source prices were given in other currencies, values reflect the referenced sources’ Euro amounts as reported in the underlying documentation.

These prices represent the market price for one unit, but the development price varies. For instance, humanoid head HRP-4C [92,93] from Japan reports an approximate of EUR 2,000,000 spent on development, and Kibo [79,80] from South Korea states that USD 9,000,000 was spent on its research and development, out of which USD 270,000 dollars were hardware costs.

## 4. Mechanical Structure

This section covers different aspects of humanoid head mechanics. Among the humanoid robots analyzed, Na’vi Shaman stands out with its impressive 42 degrees of freedom in the head, enabling an extraordinary range of motion and expressiveness. Sophia [94,95] follows closely, with 36 degrees of freedom in its head and neck, mimicking complex human movements and gestures. Albert Hubo [83,84] and Ameca [77] from Engineered Arts also have a high number of DoFs, with 28 and 25 DoFs in their faces, respectively, allowing for a high level of facial expressiveness and realism.

### 4.1. Artificial Facial Skin and Structural Materials

Humanoid robot heads are made from a range of materials carefully chosen to achieve distinct functional and aesthetic profiles. Silicone and rubber are popular materials for realistic skin simulations due to their flexibility and lifelike texture. The variety of humanoid heads shows a wide array of materials, each one carefully chosen to achieve distinct functional and aesthetic profiles. Mesmer [81,82], Sophia [94,95], HRP-4C [92,93], Geminoid F [19,20,21,22,102], Na’vi Shaman [78], and Geminoid-DK [20,21,22,34] all have silicone skin, which provides a lifelike appearance and elasticity. Waseda Flutist’s [86,87,88] vocal cord, lips, and mouth cavity are all composed of Septon thermoplastic rubber. Ameca [77] and Abel [55,56] have materials that resemble rubber and bioinspired skin.

CyberOne [96], Reachy [44,45], and Surena 4 [59], however, have bodies made of carbon fiber, aluminum, and ABS plastic. ABS plastic is frequently chosen for its weight. Carbon fiber and aluminum are commonly used for structural components due to their high strength-to-weight ratios, which is needed for mechanisms facilitating human head motions, such as the neck and eyes.

### 4.2. Neck Mechanism Mechanical Design

Most humanoid robots implement 2–3 degrees of freedom in the neck to realize pitch, yaw, and—less commonly—roll, aiming to approximate human-like head motion within compact packages.

Two archetypes dominate neck design: serial and parallel [103]. In a serial neck, actuators are stacked along the kinematic chain, each roughly aligned with one rotation axis (pitch, yaw, roll). The approach is mechanically straightforward, robust, and highly configurable from a control standpoint [70]. Representative examples include HRP-5P [98,99] and HRP-4C [92,93], with two-DoF necks (pitch/yaw), and Tino [70,71], Albert Hubo [83,84], and iCub [30,31,32] with three-DoF implementations. Despite its simplicity, serial actuation has well-known drawbacks [46]: the three rotations generally do not share a single instantaneous center, producing compound motions that can look unnatural; certain rotation combinations risk gimbal lock; and proximal actuators must carry the mass and cabling of distal ones, increasing torque demands, inertia, and volume.

**Figure 7:**
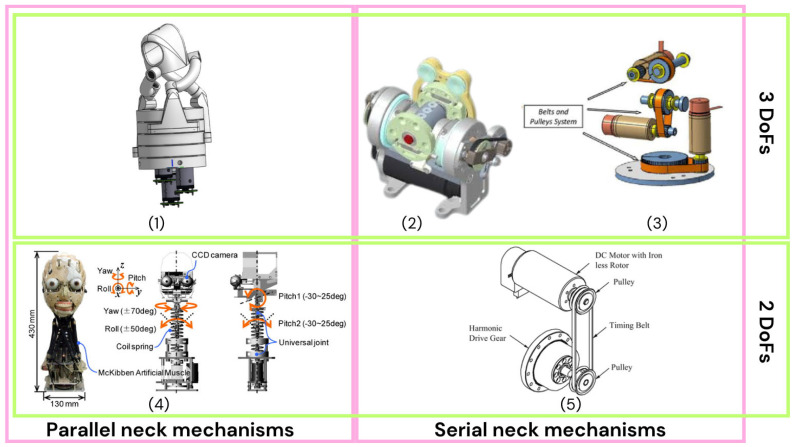
Neck mechanisms: (**1**) Reachy, (**2**) iCub, (**3**) Tino, (**4**) Saya, (**5**) HRP-4C.

A parallel neck places actuators side by side or in a distributed layout that drives the head through coupled linkages or tendons. This architecture can better approximate a human-like virtual pivot near the craniovertebral junction and reduces moving mass at the head, which helps motion naturalness and safety. However, actuator space does not map directly to Euler angles, so calibration and control are more involved, with issues such as singularities, non-intuitive coupling, and state estimation to address. Examples include Reachy [44,45] and Saya [63,64,65,66]. Examples of both categories are shown in Figure 7.

In practice, serial necks remain more prevalent in deployed systems because they are easier to package, cost-effective, and supported by mature control stacks. At the same time, there is growing interest in parallel and hybrid solutions (for example, differential or cable-driven layouts) that concentrate rotations at a single virtual pivot and lower distal inertia—attributes that improve motion naturalness and perceived comfort. The main obstacles to wider adoption are reliable calibration, avoidance of singular configurations, and controller designs that handle actuator coupling and model uncertainty without sacrificing responsiveness.

### 4.3. Eye Mechanism Mechanical Design

**Figure 8:**
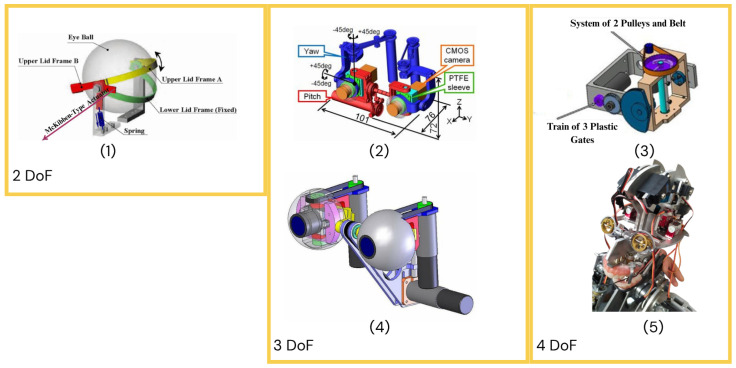
Eye mechanisms: (**1**) Saya [63,64,65,66], (**2**) iCub [30,31,32], (**3**) Tino [70,71], (**4**) KOBIAN-RII [89,90,91], (**5**) Abel [55,56].

Given the significance of eyes and their movements in creating the illusion of life in human–robot interaction, a particular focus has been placed on their design to perform believable gaze movements. A trend for humanoid heads is having eyes with three degrees of freedom, which allows them to move autonomously and resemble human eye movements. Robots such as Kismet [57,58], Dreamer [75,76,104], Waseda Flutist [86,87,88], iCub [30,31,32] KOBIAN-RII [89,90,91], and Flobi [15,16] exhibit this. Kaspar [51,52,53,54] and Saya [63,64,65,66] are able to achieve realistic results with two degrees of freedom. There are humanoid heads with more flexibility in their eye mechanism, such as Abel [55,56] and Tino [70,71], with independent eyes with four degrees of freedom total, CB2 [37,38,39] has a five-degree-of-freedom eye mechanism, and Telenoid R4 [19,20,33,34] has three degrees of freedom per eye, for a total of six degrees of freedom. These robots provide the independent eye movement needed to convey the vergence feature of the human visual system, which allows both eyes to focus on the same spot in space [70]. Some of these robots, for example, Abel [55,56], use servo motors for these mechanisms, while others, like iCub [30,31,32] and Saya [63,64,65,66], use small DC motors. Examples of eye mechanisms are displayed in Figure 8.

### 4.4. Emotional Expressive Capabilities

There are multiple layers to human–robot interaction (HRI). Each layer adds to the level of realism and complexity of a robot. Social robots use multimodal interaction methods similar to those used by humans [105], such as gesture creation (via physical embodiment), gesture recognition (via cameras or motion trackers), speech generation (via speakers), and voice recognition (via microphones).

There are currently two main methods for designing faces, each offering unique benefits and applications. The first approach, known as facial actuation, involves using motorized mechanisms to manipulate physical features to convey expressions. This technique makes it possible to precisely control facial movements, facilitating realistic interactions with people, as seen in Ameca [77], Sophia [94,95], and many others. The second method uses cutting-edge projection technology to project dynamic facial expressions onto a stationary surface, as seen in iCub [30,31,32]. This method allows for quick shifts in mood and persona by providing flexibility in both appearance and expression [6]. Versions of RoboThespian [85] are available that use both approaches. Ameca [77] and Sophia [94,95] stand out with their very human-like appearance and ability to accurately portray human expressions. Additionally, Na’vi Shaman [78] conveys human-like motions and feelings by its capability to generate exceptionally advanced facial expression. The Reachy [44,45] robot can use its antennas to convey emotions like happiness, sadness, and excitement to its audience. Other robots like Dreamer [75,76,104] can even change the color of their “ears” to express different moods.

The facial action coding system (FACS) is considered to be one of the most accepted foundations for recognizing facial expressions [106]. The FACS is a collection of facial-expression-related facial motions, also known as action units. Through the descriptive power of the FACS, facial expressions can be deconstructed and identified by a combination of action units. There are different ways of modeling emotions based on facial expressions [5]. Recent trends incorporate the usage of AI for that task, which will be further discussed in Section 7. Some humanoid heads utilize different head mechanics in order to convey human-like motions and feelings. For example, Simon’s [60,61] ear pods have two degrees of freedom, enabling the expression of emotions through subtle ear movements. Waseda Flutist’s [86,87,88] tongue has one degree of freedom, which allows it to imitate human tongue movements. Reachy’s [44,45] one-DoF antennas enable more emotional expressiveness and flexible movement.

### 4.5. Synthesis and Open Problems

Current mechanical solutions tend to privilege manufacturability and robustness over perfect anthropomorphism. Serial necks dominate because they are compact, stiff, and straightforward to package, yet their non-coincident axes yield compound motions and potential gimbal issues. Parallel or hybrid necks bring rotations closer to a virtual craniovertebral pivot and look more natural, but introduce kinematic coupling, calibration burden, and controller complexity. Eye mechanisms cluster at 2–3 DoFs per eye; adding vergence/torsion improves expressivity but increases mass, inertia, and latency—highly salient at face scale. Projection faces reduce moving parts and enable rapid persona changes, whereas mechanically actuated faces retain multi-view photometric fidelity at the cost of maintenance. Priority gaps include compact, backdrivable necks with low backlash and human-consistent kinematics; low-inertia ocular actuation that supports saccade-like profiles without overshoot; durable, repairable soft skins with stable elasticity/reflectance; and standardized, perceptually grounded metrics (e.g., FACS-aligned) for motion naturalness and expression quality over time.

## 5. Perception

**Figure 9:**
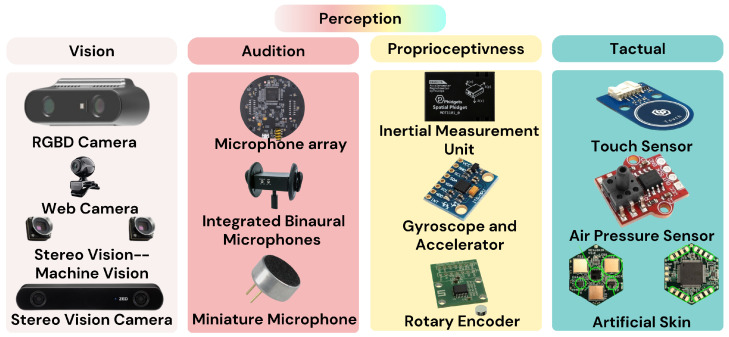
Overview of the perceptive capabilities and devices of humanoid heads.

Humanoid robot heads integrate heterogeneous sensors to approximate—and sometimes enhance—human perception, as overviewed on Table 2. Figure 9 offers a roadmap of the four principal modalities and representative devices. In what follows we examine vision, audition, proprioception, and tactile sensing, highlighting typical hardware choices, operating constraints (e.g., indoor vs. outdoor illumination, reverberation and ego-noise, latency/power limits), and integration trade-offs that shape head design and HRI performance [6]. We refer back to Figure 9 throughout; each block in the figure mirrors the structure of the subsections below.

### 5.1. Visual Perception

Cameras provide the visual input needed for scene understanding, object recognition, and navigation. Across humanoid robot heads, several complementary approaches are common, each with distinct strengths and deployment niches. Many platforms adopt RGB-D cameras—most prominently Intel RealSense—used by CyberOne [96], Sophia [94,95], and Nadine [25,26,27]. By adding per-pixel depth to RGB, these sensors simplify near-range 3D perception and manipulation. A known limitation is their reliance on active infrared patterns or time-of-flight, which makes outdoor use challenging due to sunlight interference and range constraints [107].

To address longer ranges and operation under natural illumination, many heads employ stereo camera rigs, such as those on Ameca [77], Reachy [44,45], and Octavia [100,101]. Passive stereo is robust to ambient light and scales with baseline and resolution, which is why it remains a default choice for mid- to long-range perception. Recent practice favors synchronized, global-shutter machine vision cameras that provide precise timestamps and on-board preprocessing, easing the deployment of more advanced matching and tracking algorithms on the head computer.

Some platforms add high-resolution monocular cameras—such as those on EVE [23], Olivia [28,29], and M1 [17,18]—to support fine-grained recognition, gaze estimation, and expression analysis. Beyond conventional cameras, LiDAR and other 3D scanning technologies appear on HRP-5P [98,99] and Flash [67,68]; these sensors yield accurate geometry for mapping and collision avoidance [108], but their size, power draw, and mounting constraints make head integration nontrivial [107].

Beyond binocular stereo and RGB-D, some heads can benefit from omnidirectional catadioptric stereovision, which pairs perspective cameras with curved mirrors or fisheye optics to achieve a near-360° field of view. The chief advantage of this is panoramic situational awareness with fewer large head movements—useful for multi-party HRI where speakers may enter from the periphery. Trade-offs include lower effective angular resolution, mirror-induced distortions, and a more involved calibration and rectification pipeline; compute load is typically higher than with forward-facing stereo. Compared with RGB-D, catadioptric stereo is less affected by bright ambient light and can operate outdoors, but it generally provides noisier depth at distance and requires careful photometric handling. In practice it is best used as a complement to a frontal high-resolution pair: catadioptric sensing for global context, and stereo or RGB-D for detailed interaction.

Overall, RGB-D simplifies close-range 3D perception yet struggles outdoors; stereo generalizes across lighting and distance at the cost of calibration and baseline requirements; high-resolution monocular cameras boost recognition and gaze analysis; LiDAR supplies precise geometry but is hard to package on a head; and catadioptric stereo offers panoramic context with calibration and resolution trade-offs. Effective humanoid heads combine two or more of these modalities to balance coverage, fidelity, and computational load.

### 5.2. Auditory Perception

The robots use a variety of microphone arrays to perceive and process auditory information. They are able to respond and interact verbally via speakers. Robots such as Ameca [77], Emiew 3 [12,13], Erica [19,20,34,62], and Pepper [14] use multiple omnidirectional microphones, which are often strategically placed around the head, to enable advanced sound localization and speech recognition. Reachy [44,45] and Olivia’s [28,29] sophisticated microphone arrays enable them to isolate and enhance speech signals in noisy environments, resulting in more natural and effective voice-based interactions. Some robots, such as the Geminoid-DK [20,21,22,34], have external microphones mounted near the robot to record audio for teleoperation or remote interaction. KOBIAN-RII [89,90,91] utilizes a capacitor microphone, known for its small size, lightweight build, and high sensitivity, as well as capturing an exceptional range of frequencies.

### 5.3. Proprioceptive Perception

Accurate positioning and movement tracking are required for robots to navigate their environment and perform complex tasks. To accomplish this, they mainly use rotary encoders, inertial measurement units (IMUs), and potentiometers. The Na’vi Shaman robot [78], for example, employs rotary encoders to track the absolute position of its joints, resulting in smooth and controlled movement. Flobi [15,16] and Diego-san [35,36] use IMU-based vestibular systems, which are similar to the human inner ear, to detect their orientation and movements, providing critical feedback for balance and navigation. These IMUs are placed on the parts, such as the neck and torso, that are moving, ensuring feedback information necessary for achieving mechanism stability.

### 5.4. Tactile Perception

Many robots incorporate tactile sensing in order to achieve more natural interaction with humans [5]. Erica [19,20,34,62] was outfitted with ShokacCube tactile sensors to generate natural HRI. The ability to detect height variations at the top of the soft material allows the robot to detect and respond to touch. Robots such as Pepper [14], Geminoid HI-2 [34], and NAO6 [47,48,49,50] also have capacitive sensors on their heads or bodies that detect touch and physical interactions. Diego-san [35,36], the humanoid robot, takes tactile sensing a step further, with an impressive 88 pressure sensors distributed throughout its body. This extensive network of sensors gives the robot detailed feedback on physical interactions, allowing it to better understand and respond to its surroundings and the people it interacts with. The Waseda Flutist robot [86,87,88] advances tactile sensing with a complex system of air pressure sensors, air flow sensors, optical encoders, and force sensors that allow it to precisely measure the forces and movements required to play a musical instrument.

### 5.5. Perceptual Challenges

Robot heads that resemble humans are usually made to be integrated into whole robots that resemble humans. In order to facilitate interaction with the surroundings, these heads are designed to mimic the physical traits of humans and integrate at least one main sense.

**Figure 10:**
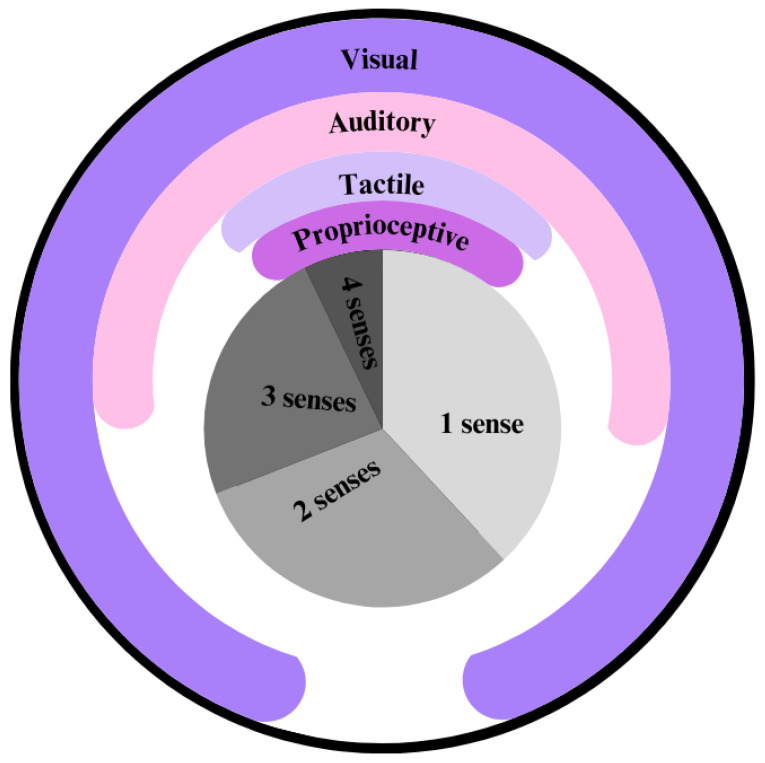
Analysis of humanoid head perceptual capabilities.

Upon examination of current robot heads, different sensors are prioritized. Most robots have only one sense implemented, with vision being the most common, as displayed in Figure 10. Features such as facial expression recognition, environment mapping, and realistic physical appearance are made possible by this. Other sensing types that are frequently integrated are auditory, proprioceptive, and tactile.

There are significant issues in implementing auditory perception in humanoid heads. Real-time separation of the sounds from multiple sources necessary for identifying the person speaking to the humanoid is challenging due to multiple factors, such as noise from the environment and the head itself (from motors). Using an array of microphones improves the recognition precision and quality at the cost of increased computational time [107].

There is no humanoid head fully mimicking all of the human senses simultaneously. This feature could be a solution to a more natural interface for a human–robot interaction. Additionally, there are potential advantages to the understudied fields of smell and temperature detection [6]. Adding a temperature sensor to a humanoid robot head can be beneficial for several reasons. It improves the robot’s ability to interact with its surroundings in a more human-like and responsive manner. For example, a temperature sensor can assist the robot in detecting and responding to changes in environmental conditions, such as entering a room with varying temperature settings. A temperature sensor in the robot’s head can play an important role in safety and maintenance [109]. By monitoring the temperature of the head, the sensor can provide early warnings of overheating, which is critical for preventing damage to sensitive electronics.

### 5.6. Synthesis and Open Problems

The sensing stack remains vision-centric: RGB-D simplifies close-range 3D perception but struggles outdoors; stereo generalizes across lighting and distance at the cost of calibration and baseline constraints; high-resolution monocular streams boost recognition and gaze analysis; LiDAR contributes precise geometry yet strains head packaging and power; and catadioptric configurations offer panoramic context with calibration and resolution trade-offs. Audition is brittle under ego-noise and reverberation, and head-mounted tactile/thermal sensing is under-deployed despite clear value for comfort and safety cues. Key gaps include audio–visual fusion robust to self-noise and lip–speech asynchrony; online calibration and uncertainty-aware state estimation tolerant of head motion; privacy-preserving, on-device perception pipelines; richer proximal sensing (tactile/thermal) for social contact; and shared HRI benchmarks that evaluate not only task success but also user comfort, trust, and longitudinal adaptation.

## 6. Computational Systems

In order to achieve intelligent behavior in complex mobile robots like humanoids, integration of a general-purpose cognitive architecture with robotics-specific software is beneficial. Creating a requirement specification for a software framework that connects cognitive architectures to robots is a complex task. Software frameworks’ intentions [110] consist of perception, action, control, and coordination functions. Initially, collecting perceptual data from various robotic sensors is necessary. Data from multiple sensors is synthesised into an overall perceptual world-view or a collection of overlapping partial world-views. Processed data is then exported to cognitive systems. Cognitive systems provide high-level or medium-level action plans, which are then transformed into detailed plans for robotic systems to execute. Determining which actions a robot should take based on its current perceptions and understanding of goals and context according to an external cognitive system or by simple rules within the robot’s control/coordination framework.

To meet these software requirements, various tools are required, including a cognitive architecture that can analyze perceptual data and recommend actions, a robot simulator, a software framework for interacting with robots, and software for generating robot movements, such as 3D graphic art or motion capture. To certain extent, if these tools are already able to work together, development will be easier. This section analyzes different computational systems used in humanoid robot heads.

### 6.1. Central Processing Unit (CPU)

The computer equipment required for humanoid heads varies greatly between models. Many use an on-board main computer, which is typically based on Intel processors such as the i7, i5, or Atom. These range from single-core to quad-core CPUs, with RAM capacities ranging from 8GB to 32GB. Examples include Sophia’s [94,95] 3GHz Intel i7 with 32GB RAM, Pepper’s [14] Intel Atom E3845, and HRP-4C’s [92,93] Intel Pentium M 1.6GHz. Some heads also use specialized processors for specific tasks, such as DSP-based controllers for joint control (CyberOne [96]), FPGAs and microcontrollers (Surena 4 [59]), or embedded TPUs (Tensor Processing Units) for local machine learning (Reachy [44,45]). FPGAs offers reconfigurability, the ability to work in parallel, time-critical processing, and optimal performance, making them well-suited for numerous applications in humanoid robotics. Digital signal processors, also known as DSPs, are specialized integrated circuits that can be configured to carry out particular kinds of computations or algorithms. They are not made to perform many, if any, of the supervisory tasks that microprocessors are normally responsible for, which sets them apart from conventional microprocessors. They are therefore far more capable of executing algorithms quickly than microprocessors or even microcontrollers. In some cases, the main computation is handled by an external control unit attached to the head, such as the Intel Core i7 computer for RoboThespian [85] or the Linux-based cluster that controls CB2 [37,38,39]. Separate computers are sometimes used for sensor processing, such as the Intel PC for sensing in Octavia [100,101] or the 500MHz Linux PC in Kismet [57,58]. Some advanced humanoid robots, such as iCub [30,31,32] and CB2 [37,38,39], use a distributed architecture with multiple computers or microcontrollers managing various functions ranging from motor control to image processing.

### 6.2. Operating System

A majority of the robots run on Linux-based operating systems, taking advantage of the flexibility, open-source nature, and real-time capabilities of these platforms. Linux-powered robots, such as CyberOne [96], EVE [23], and Surena 4 [59], can leverage a wide range of open-source tools, libraries, and simulation environments to enhance their functionality and development process. However, some robots, particularly the Geminoid series, use Windows-based operating systems, highlighting the continued relevance of proprietary platforms in certain applications.

The diversity of software and OS choices across the robots showcases the varied requirements and design approaches within the robotics industry. Pepper [14] and NAO6 [47,48,49,50] use the NAOqi operating system, a custom operating system. Developers must carefully consider factors such as performance, functionality, integration, and compatibility when selecting the appropriate software and operating system for their robot platforms. This flexibility and adaptability are essential in addressing the diverse needs of robotics applications, from industrial automation to social interaction and beyond.

### 6.3. Hardware Abstraction Layer

The software architectures of these humanoid robots often feature modular designs, with separate components handling tasks such as motion control, dialogue management, and cognitive processing. Middleware is used to bridge these components and perform hardware abstraction. This allows for easier development, testing, and deployment of new features and capabilities.

The analysis of the humanoid heads revealed that many of the robots leverage the Robot Operating System (ROS) as a common framework for sensor and actuator communication, visual perception, and control. ROS has become a widely adopted standard, enabling seamless integration and interoperability between various robot components and software modules. For example, Diego-san [35,36] uses ROS packages for sensor and actuator communication and Python, Matlab, or C++ applications for its control system. Surena 4 [59] utilizes ROS, Gazebo, and Choreonoid combination for its simulation. Robotic controls for Sophia’s [94,95] head are implemented via ROS, IK solver, PID loops, perceptual fusion, and logging/debugging tools with high-quality animation, and authoring tools for interactive performances. Reachy [44,45] leverages ROS2 packages in order to compute the kinematics of its arms and neck, obtain the camera feed, and control the autofocus on its camera’s motorized zooms. Another ROS2 package is utilized to build a server that communicates with the various ROS2 nodes and services designed for Reachy [44,45], enabling remote control of the robot even when it is not physically connected. EVE [23] utilizes ROS2 for neck, arm, and hand movement control. YARP is another common middleware choice, used first by Kismet [57,58] and then Kaspar [51,52,53,54] and iCub [30,31,32] to control and monitor the robot. YARP was developed in 2000 on a humanoid robot. However, ROS2 has proven to be a more popular choice, likely due to its large community support [111].

### 6.4. Custom Applications

Several robots utilize custom software frameworks and architectures that are tailored to their specific needs and applications, demonstrating the innovative approaches taken by robotics developers. Pepper [14] and NAO6 [47,48,49,50] use the Choregraphe software development kit (SDK) with a ROS interface for over 20 software engines, including awareness, motion, and dialogue. Their emotion engine detects how a user is feeling based on facial expressions, tone of voice, and speech, and allows the robot to respond accordingly. Some robots, like Ameca [77] and Mesmer [81,82], use specialized software frameworks like Tritium 3, which provide a comprehensive set of tools and libraries for robot control, perception, and behavior. A software architecture consists of multiple applications, each for a different robot functionality such as object tracking, facial expressiveness, conversation and different data analysis and processing. For instance, Tino [70,71] utilizes Qualisys for movement analysis. Many of the robots also incorporate a range of open-source and custom-developed software components, including computer vision libraries (OpenCV)for image processing and object tracking purposes and simulation environments (Gazebo, Choreonoid) for testing head actuators. These applications are often implemented in programming languages like Python, C++, and Java. Moreover, AI is an important point of development for humanoid robots.

### 6.5. Communication Protocols

The humanoid robots discussed use a variety of communication protocols to facilitate the exchange of data and control signals. Ethernet connectivity is a popular choice, as seen in implementations in NAO6 [47,48,49,50], Reachy [44,45], Sophia [94,95], Zeno [40,41], Pepper [14], and the RJ45 interface in Ameca [77]. Some of these robots have wireless communication capabilities besides Ethernet connectivity, such as Wi-Fi and Bluetooth. These features allow humanoid creations more flexibility and autonomous operation, as demonstrated by Reachy [44,45], Sophia [94,95], and Zeno [40,41] for Wi-Fi connectivity and NAO6 [47,48,49,50] and Pepper [14] for the Bluetooth connectivity. For faster data transfer, usage of USB connectivity is still prominent, despite its physical drawbacks due to cable length [112]. Some of the robots using this connectivity method include Kaspar [51,52,53,54], Ameca [77], Reachy [44,45], and KOBIAN-RII [89,90,91]. Serial protocols such as RS-232C and RS-485 are used for more specialized needs, as also seen with KOBIAN-RII [89,90,91]. Furthermore, the demand for flexibility, outstanding performance, and reliability [113] is met by incorporating the EtherCAT protocol into Dreamer’s [75,76,104] architecture.

### 6.6. Power Sources

Humanoid robots use a variety of power sources to function, each with advantages and disadvantages. Overall, the choice of power source for humanoid robots is determined by factors such as the robot’s size, weight, runtime requirements, and intended use, with each power solution providing trade-offs in terms of portability, runtime, and complexity. For instance, battery-powered robots offer portability and extended operational runtime. However, they may be constrained by weight and the necessity of frequent recharging. Conversely, tethered power supplies provide uninterrupted energy, yet they restrict the robot’s mobility. Many robots, including Pepper [14], whose runtime is 12 h, EVE [23], whose runtime is 4 h, CyberOne [96], whose runtime is 2.5 h of operation, and KOBIAN-RII [89,90,91], whose runtime is 20 min, run on lithium-ion battery packs, which provide a compact and portable power source. Some robots, such as Reachy [44,45], whose runtime is 10 h, use lithium-ion batteries with battery management systems (BMSs) to ensure safety and extended runtime. Other robots, such as Ameca [77] and Mesmer [81,82], use external 24VDC and 200W power supplies, which provide a consistent power source but limit the robot’s mobility. Compressed air is another power source used by some humanoid robots, such as Erica [19,20,34,62] and Geminoid HI-2 [34], which drive pneumatic actuators in conjunction with electrical motors and control systems.

### 6.7. Synthesis and Open Problems

Most heads run Linux with ROS/ROS2 or YARP for modularity, layered over heterogeneous compute (CPU/GPU/TPU/FPGA) and fieldbuses such as Ethernet/EtherCAT for timing-critical loops. The core tension is flexibility vs. determinism: complex graphs, variable QoS, and multiprocess IPC complicate end-to-end latency guarantees, time synchronization, and fault containment. Open problems include deadline-aware composition of perception–planning–control pipelines; resource-aware scheduling across heterogeneous accelerators within tight power envelopes; privacy-first on-device ML that minimizes cloud dependence; lifecycle management with safe rollback and signed updates; and built-in introspection/health monitoring so that failures degrade gracefully rather than abruptly.

## 7. Role of AI in Humanoid Robotics

AI is an important point of development for creating intelligent and emotionally aware humanoid robots. Initially, the design of the robot is critical, with the goal of creating an efficient machine that conserves energy and remains balanced while in operation. This process refines the robot’s structure and functionality using nature-inspired optimization techniques [114]. Several humanoid robots stand out for their use of advanced artificial intelligence technologies, and this section analyzes ways AI is being integrated into humanoid robot heads. Sophia [94,95], developed by Hanson Robotics, uses the Hanson AI SDK to empower the robot with intelligent and responsive behaviors. The Hanson AI SDK contains a variety of perception and control commands. Some of its features include face tracking, recognition, expressions, gestures, reactions to perception (tracking, imitation, and saccades), and simulations using Gazebo, Blender, and Unity. Pepper [14] elevates emotional intelligence with its innovative “emotion engine”—a system that dynamically assesses a user’s facial expressions, vocal tone, and speech patterns to tailor interactions accordingly. Erica [19,20,34,62] and EVE [23], among others, use Java and Python applications to enable AI-powered perception, decision-making, and natural language processing. These are the main humanoids that utilize AI as part of their control architecture.

### 7.1. Advanced Navigation Systems

Once the head is designed and integrated, the robot continuously acquires multimodal data from on-board cameras and other sensors. Visual streams follow a standard pipeline—image acquisition and synchronization, preprocessing (e.g., rectification and denoising), segmentation and edge/feature extraction, and learning-based detection and classification—to infer objects, people, and scene layout [115].

Because measurements from individual sensors are noisy, partial, and sometimes contradictory, extracting reliable information is nontrivial; the challenge compounds when modalities are combined [116]. Humanoid heads therefore employ multi-sensor fusion schemes that explicitly handle imprecision and uncertainty, with fuzzy-set methods being a common choice in this context [117]. At the behavior level, fuzzy logic and neural networks can adapt gaze, head posture, and gait parameters in response to environmental cues, supporting smoother operation across varied conditions [115]. For navigation, a Fuzzy Markov Decision Process (FMDP) has been applied to NAO to compute collision-free paths under uncertainty [47,48,49,50], providing a principled decision-making formalism when observations and rewards are imprecise [118].

Another AI navigational technique implemented on NAO [47,48,49,50] is the hybridization of the Dynamic Window Approach (DWA) and the Teaching–Learning-Based Optimization (TLBO) technique [119]. The input is based on the location of obstacles and the target. The parameters are provided to the DWA technique [120], which decides the optimum velocity. Robots utilizing ROS can benefit from this AI navigation algorithm configuration standalone, as ROS provides a package that operates the robot navigation based on this algorithm [121,122]. The intermediate result is feed to the TLBO technique, which operates based on the teacher phase and the learner phase [123]. This hybridization provides an optimum angle to take a turn and avoids the obstacles while moving towards the target.

### 7.2. Speech Generation and Voice Recognition

Speech generation and voice recognition are crucial aspects of human–robot interaction, as they facilitates verbal communication between humans and robots. The technology commonly used for different aspects of verbal communication in human–robot interaction, such as question-answering [105], is natural language processing(NLP). Natural language processing is a machine learning technology for the interpretation, manipulation, and understanding of human language. Natural Language Generation (NLG) is a subfield of NLP responsible for the generation of natural language responses to humans.

Sophia [94,95] has impressive language processing and conversational abilities. It can hold conversations, understand and respond to questions, and even show a variety of emotional expressions. HRP-4C [92,93] elevates speech generation with its capability to sing and create melodious sounds. Its advanced speech synthesis capabilities allow it to produce human-like singing voices, making it an excellent platform for entertainment and performance applications. Cyber One is exceptional in regard to the range of sounds it is able to identify, recognizing 85 types of environmental sounds and 45 classifications of human emotion.

### 7.3. Large Language Models

The combination of large language models and robotic systems has created an entirely novel approach in human–robot interaction, providing outstanding capabilities in natural language understanding and task execution [124]. Large language models (LLMs) are deep learning models that have a large number of parameters and are trained unsupervised on large amounts of text. While the exact parameter counts of state-of-the-art models such as GPT-4 have not been publicly disclosed, many contemporary LLMs are multimodal and can process both text and images [125]. LLMs serve as the robot’s brain, integrating knowledge, memory, and reasoning to plan and execute tasks [126] intelligently.

The introduction of LLMs has revolutionized NLP over the last decade. This has resulted in a rise of innovation and creativity in the field. These cutting-edge models, powered by massive amounts of data and complex neural network architectures previously used in other areas of modeling, have demonstrated unparalleled capabilities in understanding, generating, and manipulating human language. From the renowned GPT to its successors, these massive language models have demonstrated their abilities in various tasks, including text production, translation, sentiment analysis, and question-answering. In addition, LLMs offer more diverse conversation content and personalized interaction experiences. Robots can customize interactions with LLMs to meet user preferences and needs. This enhances user satisfaction and interactions. Using large models with natural language processing and emotion analysis improves robots’ understanding of human emotions and intentions, leading to more natural and intelligent interactions with humans [126].

### 7.4. Emotional Intelligence

Robots’ emotional intelligence is developed using algorithms based on human emotions. These tools allow the robots to recognize and respond to emotional cues, improving their ability to interact with humans and manage social situations effectively. The “Loving AI” project [95] aims to create software that allows humanoid robots, such as Sophia [94,95], to interact with humans in a loving and compassionate manner, promoting self-understanding and transcendence. This project has improved the OpenCog framework and Hanson humanoid robot software in critical areas using deep neural networks. This AI-driven, audio–visual, interactive android technology enhances human development through subjective and objective measures [127]. This allows the system to understand and respond appropriately to users’ emotional states, which improves interactive bonding. Furthermore, software has been created to enable robots or avatars to recognize and mimic human facial expressions and vocal qualities. Furthermore, significant effort has been made to improve the motivational and emotional aspects of the OpenCog system. The OpenPsi framework has been extended to include these aspects, which are based on Personality Systems Interaction (PSI) theory [128] and the Component Process Model (CPM). PSI theory aims to model the complex relationship of emotional, motivational, and cognitive processes [128]. The CPM emphasizes continuous monitoring of the organism’s environment and recognizes that emotional reactions are complex, multifaceted processes that cannot be described using verbal labels [129]. The comprehensive approach that combines two theories aims to provide the system with a better understanding and expression of human-like emotions, thereby improving the overall user experience.

### 7.5. Synthesis and Open Problems

AI now spans classical controllers and learned components for dialogue, gaze and expression timing, and decision-making under uncertainty; large language models broaden interaction but introduce hallucination risk, timing drift, and privacy concerns. Central challenges are grounding high-level policies in verifiable sensorimotor affordances, aligning micro-timing across speech, lip motion, gaze, and blinks, and adapting to users with minimal data while preserving safety. Priority directions include robust grounding and tool-use for head-level tasks; few-shot, on-device personalization that respects consent and data minimization; evaluation beyond task success to include comfort, trust, and long-term adherence; and transparent uncertainty/intent communication that keeps humans in the loop without eroding the sense of naturalness.

## 8. Ethical, Sociological, and Economic Implications

Humanoid robot heads are increasingly considered for healthcare and education, where they promise consistent cues, repeatable practice, and engaging interfaces. In dementia care and rehabilitation, heads can scaffold routines and social stimulation; in educational settings and autism interventions, predictable gaze, expressions, and timing may lower interaction barriers. Social acceptance, however, hinges on context-appropriate design and behavior. More stylized embodiments often fare better in public spaces, whereas highly realistic heads demand tighter control of micro-movements and timing to avoid discomfort. Deployments should be framed as assistive—with clinician/educator oversight—rather than as replacements for human care, and should include transparent communication of capabilities, limitations, and escalation paths to a human-in-the-loop.

Privacy and security require special attention because head-mounted microphones and cameras can capture sensitive audio–visual and biometric data at close range. A privacy-by-design approach is essential: purpose-limited capture tied to an explicit task; on-device processing by default with opt-in cloud services only when strictly necessary; data minimization and short, configurable retention; conspicuous recording indicators and physical hard-mute controls for audio/video; role-based access for caregivers, clinicians, or teachers; and audit logs for dataflows and model updates. Safety measures should address spoofing and remote compromise (e.g., signed updates, least-privilege networking), and include failure modes that degrade gracefully (for example, neutral facial posture and relaxed neck on fault).

Economic considerations extend beyond sticker price. As shown in Figure 6, price levels vary widely across countries and platforms; consequently, procurement decisions should be based on total cost of ownership: hardware, spares, consumables, software licenses/updates, integration, maintenance, operator training, and expected lifetime. Cost-effectiveness improves when heads can be repurposed across multiple tasks (telepresence, coaching, triage, social engagement), when open standards reduce vendor lock-in, and when on-device processing lowers recurring cloud costs and mitigates compliance burdens.

In practice, we recommend a deployment blueprint that is privacy-first and outcome-oriented: define the task and data strictly in advance; default to local processing with clear user consent and visible controls; document retention and access policies; monitor comfort, trust, and adherence longitudinally rather than relying on single-session metrics; keep a qualified human supervisor in the loop; and evaluate value using TCO rather than upfront price alone. These guardrails support responsible use of humanoid heads while aligning technical capabilities with social expectations in clinics and classrooms.

## 9. Conclusions

The mechatronics aspects that are essential to the development of humanoid robot heads have been thoroughly reviewed in this review. These aspects encompass sensors, software, processors, communication protocols, applications, and related challenges. Utilizing an extensive dataset consisting of 42 humanoid robot heads from 1998 to 2022, this analysis provides insightful information about the development and state of this field today.

The challenge of creating a humanoid robot head at the intersection of robotics, artificial intelligence, and cognitive science is complex and formidable: it must accurately mimic human facial expressions and physical appearance while authentically replicating all human senses. The inability of present AI technologies to successfully synthesize the complexities of human behavior and perception, despite significant advancements in each of these areas, presents a barrier to the development of genuinely lifelike humanoid robots. A deep navigation of human psychology and aesthetics is necessary to explore the fine line between achieving realism and avoiding the unsettling effects of the uncanny valley. A full sensory system integration into a robotic head adds to the complexity. There is no humanoid currently perfectly capturing all of the human senses at once. Overcoming these obstacles requires multidisciplinary cooperation. Creative solutions that bridge the gap between artificial and human intelligence must be developed using knowledge from computer science, psychology, neuroscience, and engineering. While this investigation has provided a thorough understanding of the electrical and mechanical complexities involved, it is critical to recognize that these components represent only one aspect of the larger effort required to realize a comfortable human–robot interface. Successful integration necessitates collaboration between electrical, mechanical engineering, materials science, and cognitive science to ensure not only a lifelike appearance but also genuine intelligence. Furthermore, this review revealed an absence of literature focusing specifically on the software aspect of humanoid robot heads. This observation highlights a gap in existing research and emphasizes the need for future studies to delve deeper into the software algorithms, AI techniques, and cognitive models that drive these robots’ functionality. Addressing this gap will help us gain a better understanding of humanoid robots and develop them more effectively. Ethical issues [130] surrounding the development and application of humanoid robots should be considered also, as they are especially relevant in situations like caregiving, companionship, and customer service.

Looking ahead, interdisciplinary collaboration is critical for overcoming persistent challenges and advancing the field of humanoid robotics. By encouraging dialogue and collaboration across disciplines, we can leverage collective expertise to accelerate the integration of humanoid robots into various aspects of human life, contributing to advances in healthcare, education, entertainment, and other areas.

## Figures and Tables

**Table 1:** Search sources, terms, criteria, and dates (at a glance).

Item	Details
Databases	IEEE Xplore; ACM Digital Library; Scopus; Web of Science Core Collection; ScienceDirect; SpringerLink; PubMed; arXiv (screened)
Conferences	ICRA; IROS; Humanoids; HRI; RO-MAN (proceedings screened)
Time window/last search	January 1998–December 2022; final search on 31 December 2022
Representative terms	“humanoid head”, “android head”, “robot head”, “robotic face”; mechanics, “neck mechanism”, “eye mechanism”, FACS, actuator*, “artificial skin”; vision, stereo, RGB-D, LiDAR, catadioptric, microphone*, IMU, tactile; ROS/ROS2, middleware, YARP, “real-time”, EtherCAT; HRI, “uncanny valley”
Boolean pattern (example)	(“humanoid head” OR “android head” OR “robot head” OR “robotic face”) AND (mechanics OR “neck mechanism” OR “eye mechanism” OR FACS OR actuator OR “artificial skin”) AND (vision OR stereo OR RGB-D OR LiDAR OR catadioptric OR microphone OR IMU OR tactile) AND (ROS OR ROS2 OR middleware OR YARP OR “real-time” OR EtherCAT) AND (HRI OR “human-robot interaction” OR “uncanny valley”)
Inclusion criteria	(i) humanoid/android head or humanoid platform with a documented head module; (ii) human-like appearance; (iii) at least one head-mounted perceptual modality and/or a head-specific mechanical design relevant to HRI; (iv) technical details on mechanics, sensing, computation, or control/HMI; English source preferred when duplicates existed
Exclusion criteria	Headless platforms; purely artistic installations without reproducible technical description; non-human-like shells with no HRI relevance; no head-mounted perception; duplicates
PRISMA counts	Retrieved: n=276; after contextual screening: n=97; after appearance filter: n=56 (excluded 41); after functionality filter (≥1 sense): n=42 (excluded 14); see Figure 4.

**Table 2:** Sensor information equipped in humanoid robot heads.

Robot	Vision	Audition	Proprioceptive	Tactile
CyberOne	Intel RealSense D455 RGB-D camera	–	–	–
Ameca	Binocular eye-mounted cameras; chest camera	Embedded microphones	–	Current sensing on all servos
Abel	Integrated camera	Integrated binaural microphones	–	–
Surena 4	RealSense D435 camera	–	–	–
HRP-5P	Stereo vision; LiDAR; 3D sensors	–	–	–
Na’Vi Shaman	–	–	Rotary encoders for absolute positioning	–
Mesmer	Cameras; depth sensors; LiDAR	Microphones	–	–
Sophia	Intel RealSense camera	External USB microphone; audio localization array	IMU	–
Emiew 3	CCD camera	Microphone array	–	–
Tino	Two cameras	–	–	–
Erica	Two CMOS cameras	Two microphones	–	–
Pepper	Two HD 5-MP cameras (mouth/forehead); 3D sensor (behind eyes)	Four microphones	–	3 touch sensors
KOBIAN-RII	CMOS camera	Capacitor microphone	IMU	–
Roboy	Two cameras	One microphone	–	–
Telenoid R4	–	Two microphones	–	–
Nadine	Microsoft Kinect V2 RGB-D camera; web cameras	Microphone	–	–
Dreamer	Stereo cameras on eyes	Microphones	–	–
Flash	Microsoft Kinect; Logitech QuickCam Sphere camera	–	Analog Devices inertial system	–
Diego-San	Two Point Grey Dragonfly2 cameras	Two microphones	Two IMUs	38 potentiometers; 88 pressure sensors
Geminoid F	External webcam	–	–	–
HRP-4C	CCD camera	–	–	–
Simon	Point Grey FireflyMV CMOS cameras	–	–	–
Saya	CCD camera	Microphones	–	–
Zeno	Two 720p, 30 fps HD cameras	Microphones	Three-axis gyroscope; three-axis accelerometer; compass	–
iCub	Stereo cameras	Microphones	Gyroscopes; accelerometers	–
Kismet	Four CCD cameras	Two microphones + one for person speaking	–	21 encoders

## Data Availability

Data derived from public domain resources.
